# Long-term exposure to hypoxia inhibits tumor progression of lung cancer in rats and mice

**DOI:** 10.1186/1471-2407-11-331

**Published:** 2011-08-03

**Authors:** Lunyin Yu, Charles A Hales

**Affiliations:** 1 Pulmonary and Critical Care Unit, Department of Medicine, Massachusetts General Hospital, Harvard Medical School, Boston, MA 02114, USA

**Keywords:** hypoxia, tumor growth, metastasis, A549 lung cancer cells, Lewis lung carcinoma, HCT116 colon cancer cells, animals

## Abstract

**Background:**

Hypoxia has been identified as a major negative factor for tumor progression in clinical observations and in animal studies. However, the precise role of hypoxia in tumor progression has not been fully explained. In this study, we extensively investigated the effect of long-term exposure to hypoxia on tumor progression *in vivo.*

**Methods:**

Rats bearing transplanted tumors consisting of A549 human lung cancer cells (lung cancer tumor) were exposed to hypoxia for different durations and different levels of oxygen. The tumor growth and metastasis were evaluated. We also treated A549 lung cancer cells (A549 cells) with chronic hypoxia and then implanted the hypoxia-pretreated cancer cells into mice. The effect of exposure to hypoxia on metastasis of Lewis lung carcinoma in mice was also investigated.

**Results:**

We found that long-term exposure to hypoxia a) significantly inhibited lung cancer tumor growth in xenograft and orthotopic models in rats, b) significantly reduced lymphatic metastasis of the lung cancer in rats and decreased lung metastasis of Lewis lung carcinoma in mice, c) reduced lung cancer cell proliferation and cell cycle progression *in vitro*, d) decreased growth of the tumors from hypoxia-pretreated A549 cells, e) decreased Na^+^-K^+ ^ATPase α1 expression in hypoxic lung cancer tumors, and f) increased expression of hypoxia inducible factors (HIF1α and HIF2α) but decreased microvessel density in the lung cancer tumors. In contrast to lung cancer, the growth of tumor from HCT116 human colon cancer cells (colon cancer tumor) was a) significantly enhanced in the same hypoxia conditions, accompanied by b) no significant change in expression of Na^+^-K^+ ^ATPase α1, c) increased HIF1α expression (no HIF2α was detected) and d) increased microvessel density in the tumor tissues.

**Conclusions:**

This study demonstrated that long-term exposure to hypoxia repressed tumor progression of the lung cancer from A549 cells and that decreased expression of Na^+^-K^+ ^ATPase was involved in hypoxic inhibition of tumor progression. The results from this study provide new insights into the role of hypoxia in tumor progression and therapeutic strategies for cancer treatment.

## Background

Cancer is a major public health problem in the United States [1] and many other countries in the world [2-4]. One in 4 deaths in the United States is due to cancer [1]. Although much effort has been made and the overall cancer incidence rate has decreased in the most recent time period, a total of more than 1.5 million new cancer cases and more than half million deaths from cancer are projected to have occurred in the United States in 2010, of which lung cancer is the leading cause of cancer death in both men and women [5].

Hypoxia which is often seen in solid tumors [6,7] has been identified as a major negative prognostic factor [8-19], because decreased availability of oxygen in the tumor increases treatment resistance and favors tumor progression and metastasis [15,20]. Development of hypoxia in human solid tumors is due to rapid proliferation of tumor cells and the relative deficiency of blood distribution in the tumor mass [15,20], resulting in low oxygen levels in tumor cells, so-called hypoxic cells, located at a distance from the blood vessels [20]. Tumor hypoxia is an important factor in tumor biology which is associated with angiogenesis, tumor cell aggressiveness, metastasis and local recurrence [8,10,11,21].

In addition to observations from clinical data, animal experiments have investigated the relationship between tumor hypoxia and cancer progression. Investigators have found that metastasis occurred significantly more often in primary tumor tissues with high hypoxic fractions [22], and that the more hypoxic cells present in tumor tissue, the more lung and lymph node metastasis occurred in mice bearing tumor [23]. They also found that acute hypoxia influenced metastasis to a greater extent than chronic hypoxia [23]. Studies have shown that pretreatment of the cells with low oxygen induced maximal lung metastasis [24] and that tumor hypoxia was correlated with the number of metastatic lesions, but not with tumor volume [25]. Studies have shown that exposure to hypoxia significantly increased lung metastasis in mice [26,27]. These studies also showed that exposure to hypoxia significantly increased the number of positive lymph nodes in mice, but not in lung metastasis nodule [27,28]. A recent report showed that systemic hypoxia promoted prostate cancer growth in mice [29]. However, another study found no significant change either in primary tumor growth or in lung metastasis in a transgenic mouse breast cancer model after exposing the mice to hypoxia [30]. Therefore, in spite of much work done in different laboratories, the precise role of hypoxia on tumor progression is far from being completely understood [31].

Recently we unexpectedly found that hypoxia (10% O_2_) strongly inhibited tumor growth of lung cancer in nude rats. We therefore extensively investigated the role of hypoxia in tumor progression in this study. We hypothesized that hypoxia may not be a factor favoring tumor progression of lung cancer.

## Methods

### Cancer cells

Human non-small cell lung carcinoma cell line A549 cells (A549 cells) and Lewis lung carcinoma cell line LLC1 cells (established from the lung of a C57BL mouse bearing a tumor) were purchased from ATCC (American Type Culture Collection), Manassas, VA. The A549 cells and LLC1 cells were grown in F-12K Medium and in Dulbecco's Modified Eagle's Medium (DMEM) respectively. In addition, human colon cancer cell line HCT116 cells (HCT116 cells) and McCoy's modicum were also purchased from ATCC.

### Animals

NIH nude rats (Cr:NIH-rnu), weighing ~ 150 g, were obtained from the National Cancer Institute at Frederick (NCI-Frederick, Frederick, MD). Male BABL/c nude mice, 6 weeks old, were obtained from Charles River Laboratories (Wilmington, MA). C57BL/6 mice, 8 to 10 weeks old, were supplied by Jackson Laboratory (Bar Harbor, Maine). These animal experiments were approved by the ethics committee for animal care of the Subcommittee on Research Animal Care at Massachusetts General Hospital and had followed the USA Institutional Animal Care and Use Committee (IACUC) guideline.

### Hypoxia exposure

Animal exposure to hypoxia was performed as described previously for our hypoxic pulmonary hypertension studies [32,33]. Briefly, the animals were weighed and placed in a hypoxia chamber or exposed to normoxia for a designed time. Oxygen concentration was maintained by the flow rates of compressed air and nitrogen. Cage concentration of O_2 _was checked daily. The cages were opened once a day to reduce CO_2 _concentration. The animals were supplied with food and water *ad libitu *during the experiments.

### Subcutaneous xenograft models of lung cancer

Each animal was inoculated subcutaneously on the flank with a single dose of 2 × 10^7 ^A549 cells in 200 μl of phosphate-buffered saline solution, cell viability > 95%. No Matrigel was used for this tumor cell injection. At different time points (day 1, day 4 and day 7) after cancer cell injection, the animals were randomly divided into normoxic and hypoxic groups for hypoxia exposure for desired time. At end of the experiment, animals were sacrificed and tumors were harvested. The same procedures were performed for colon cancer using HCT116 cells in this study.

### Orthotopic rat model of lung cancer

A549 cells were orthotopically injected into the left lung of rats according to published methods [34,35]. Briefly, male nude rats were anesthetized with isoflurane by using a rodent anesthesia machine. The rats were placed in the right lateral decubitus position and an incision of the skin was made to visualize the left lung. A single dose of 2 × 10^7 ^A549 cells in 100 μl of phosphate-buffered saline solution was injected into the left lateral lung of the rats. Followed closure of the incisions with sutures, the rats were turned to the left lateral decubitus position until they had fully recovered at which time the rats were returned to their cage. Four days after cancer cell injection, the rats were placed in the hypoxia chamber. After exposure to hypoxia for 10 days, the animals were removed from the hypoxia chamber and tumors were harvested. At the same time, mediastinal lymph nodes were collected for evaluating the effect of long-term exposure to hypoxia on lymphatic metastasis.

### Lung metastasis model of Lewis lung carcinoma

LLC1 cells (1 × 10^7 ^cells) were injected subcutaneously into the left flank of male C57BL/6 mice according to the method described previously [26,36]. On day 4 after the tumor cell injection, the tumor volume was measured and then the animals were randomly divided into normoxic and hypoxic groups. The mice for the hypoxic group were placed in hypoxia chamber for 17 days. The lung metastasis of Lewis lung carcinoma was assessed according to methods described previously [26,36]. Briefly, the lungs were removed from sacrificed mice and filled with Bouin's fixative solution through trachea and then immersed in the fixative. After 48 hours, the number of metastatic foci on the lung surface was counted with a dissecting microscope.

### Hypoxia-pretreatment and tumor progression in mice

In order to investigate the effect of hypoxia on cultured cancer cells *in vitro *and to determine the effect of hypoxia-pretreatment on tumor growth in animals, we carried out the following study. After seeding and growth for a few hours under normoxia allowing to attach to the bottom of flask, A549 cells were cultured in a cell culture hypoxia chamber (0.5%O_2_/5%CO_2_/N_2 _balance) for seven days and then harvested for cell proliferation assay, cell cycle analysis and biological analysis. At the same time, other harvested cancer cells were injected into nude mice subcutaneously (5 × 10^6^/per mouse). After three weeks under normoxia, the mice were sacrificed and tumors were harvested. In addition, HCT116 colon cancer cells were inoculated into mice subcutaneously in this study with the same conditions and procedures as used for the A549 cells to compare the effect of chronic hypoxia on other type of cancers.

### Measurement of tumor growth and evaluation of tumor weigh

For the subcutaneous xenograft model, we used a tumor growth curve based on tumor size for evaluation of tumor growth during the period of experiment. The tumor size (in cubic millimeters) was calculated according to a formula (L × S^2^) × 0.5 (L = long diameter of tumor, S = short diameter of tumor) [37]. In addition, on the last day of the experiment, animals were sacrificed with 200 mg/kg of pentobarbital and used immediately for determination of tumor weight and pathology as well as for biological analysis. Tumor weight was determined by weighing the wet tumor. Part of the tumor was fixed in 10% natural buffered formalin, embedded in paraffin, sectioned at 4-6 μm and stained with H & E or with immunohistochemistry for pathological evaluation. The rest of the tumor tissue was frozen immediately in liquid nitrogen for biological analysis.

### Immunohistochemistry

Immunohistochemical staining with Ki67, a cell proliferation marker, was performed to analyze cell proliferation. A percentage of Ki-67 positive cells expressed as Ki67 proliferative index was estimated by calculating the ratio of Ki-67 expressing cell nuclei to the total number of cell nuclei in tumor tissues. TUNEL assay was conducted by using TdT FragEL DNA Fragmentation Detection Kit (Oncogene Research Products, San Diego, CA), following the manufacturer's protocol. An apoptotic index was determined by a blinded investigator by counting the ratio of apoptotic cells to total cells in the tissue section. In addition, CD31 antibody (Abcam, Cambridge, MA) was used to detect tumor microvessel density. The density was quantified by calculating the number of CD31 positive microvessels under microscope.

### Mitosis analysis in tumor tissues

The number of mitotic cells in tumor tissues was counted under H & E stained slides by a blinded pathologist. A mitosis index was calculated and expressed as mitotic cell number/total cell number × 100.

### Western blot analysis

Antibodies included HIF1α and Na^+^-K^+ ^ATPase α1 (Sigma) and HIF2α and ß-actin (Santa Cruz Biotechnology). Total proteins were isolated from tumor tissues and cancer cells and Western blot was performed following our previous work [37,38]. Briefly, homogenized tissues were incubated on ice for 30 minutes in lysis buffer and then centrifuged at 14,000 rpm for 10 minutes at 4°C. The supernatant was removed and saved. Supernatants were stored at -80°C until analysis. The protein concentration of the lysate was determined by the Bio-Rad protein assay (Bio-Rad Laboratories, Hercules, CA). Protein samples were electrophoresed on SDS-polyacrylamide gel and then transferred to polyvinylidene difluoride (PVDF) membranes (Millipore Corporation, Bedford, MA). After an overnight incubation with PBS with 10% nonfat milk, the membrane was incubated with primary antibody for 1 hr at room temperature or overnight at 4°C. The membrane was washed with buffer and then incubated with a horseradish peroxidase linked secondary antibody for 1 hr at room temperature. After being washed with buffer, the signals were detected using an enhanced chemiluminesence (ECL) Western blot detection kit, Western Lightning (PerkinElmer Life Sciences, Boston, MA), and visualized by exposure to X-ray film. Quantification of protein expression was performed using NIH 1.61 image software.

### Reverse transcription-polymerase chain reaction (RT-PCR)

Total RNA was extracted from tumor tissues using TRIzol reagent (Invitrogen, Carlsbad, CA) and RT-PCR was performed following our previous work [37-39]. Briefly, total RNA (3 *μ*g) was used to carry out RT-PCR to measure mRNA expression with Ready-To-Go Your-Prime First-Strand Beads (Amersham Biosciences UK Limited, Little Chalfront, Buckinghamshire, England) for reverse transcription and Platinum PCR SuperMix reagents (Invitrogen, Carlsbad, CA) for PCR according to the manufacturer's instructions respectively. The primer pairs for HIF1α and for the housekeeping gene glyceraldehyde-3-phosphate dehydrogenase (GAPDH) were purchased from Sigma Genosys, Woodlands, TX. After RT-PCR, each sample was run in agarose gel (15.0 g/L) electrophoresis to ensure that the right-size product was amplified in the reaction. Bands were visualized using ethidium bromide and the gels were photographed under UV light. Quantification of RT-PCR products was performed using NIH 1.61 image software.

### Statistical Analysis

Statistics were performed using the computer program Statview (SAS Institute Inc., Cary, NC) with t-Test. All values were expressed as the mean ± SEM. Significance was set at p < 0.05.

## Results

### Exposure to hypoxia inhibited lung cancer tumor growth in nude rats

**Figure 1:**
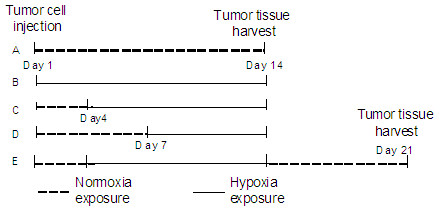
**Experimental Protocols**. (A) Animals in normoxia for 14 days after tumor cell injection. (B) Animals in hypoxia for 14 days after tumor cell injection. (C) Animals in normoxia for 4 days after tumor cell injection and then in hypoxia for 10 days. (D) Animals in normoxia for 7 days after tumor cell injection and then in hypoxia for 7 days. (E) Followed in normoxia for 4 days after tumor cell injection, animals were placed in hypoxia for 10 days and then returned to normixa for another 7 days.

**Figure 2:**
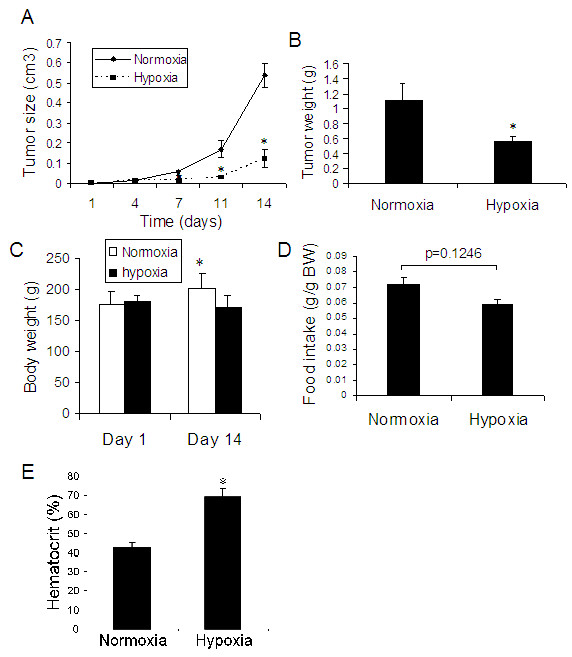
**Effect of hypoxia to hypoxia for 14 days on tumor growth of lung cancer in rats**. After cancer cell injection, animals were exposed to hypoxia (10% O_2_) for 14 days (Figure 1B). (A) Tumor growth curve, (B) Tumor weight, (C) Body weight, (D) Food intake and (E) Hematocrit. * p < 0.05 as compared with normoxia. n = 6 for each group.

After injection with A549 cells subcutaneously, rats were exposed to hypoxia for 14 days (Figure 1B). We found that exposure to hypoxia significantly decreased the growth of tumor from A549 cells (lung cancer tumor) (Figure 2), showing decreased tumor size during hypoxia exposure (Figure 2A) and reduced tumor weight (Figure 2B) as compared with the animals under normoxia. Hypoxic animals showed a decrease in body weight (Figure 2C) and in food intake (Figure 2D). Hematocrit was significantly increased in the hypoxic animals (Figure 2E).

**Figure 3:**
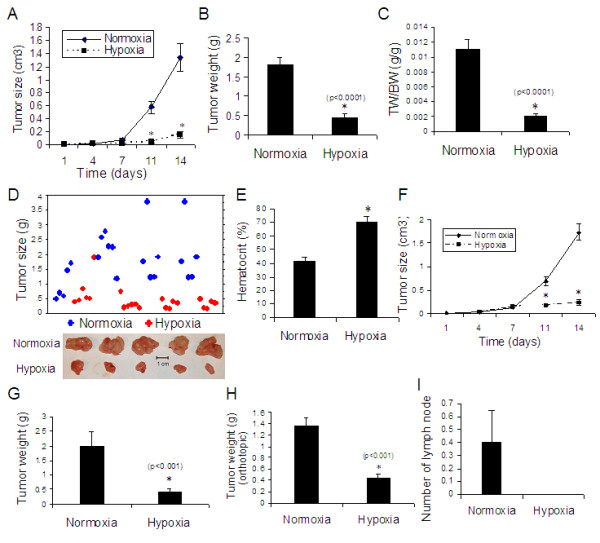
**Effect of hypoxia to hypoxia for 10 days or 7 days on tumor growth of lung cancer in rats.(A to E) Exposure to hypoxia for 10 days in xenograft model (Figure 1C)** : **(A)** Tumor growth curve, **(B)** Tumor weight, **(C)** ratio of tumor weight to body weight, **(D)** Distribution of tumor size (upper panel) and representative tumor samples (lower panel), **(E)** Hematocrit. *p < 0.05 as compared with normoxia. n = 21 for normoxia group and 22 for hypoxia group. **(F & G) Exposure to hypoxia for 7 days in xenograft model (Figure 1D)**: **(F)** Tumor growth curve and **(G)** tumor weight. n = 5 for each group. *p < 0.05 as compared with normoxia. **(H & I) Exposure to hypoxia for 10 days in orthotopic model (Figure 1C)**: **(H)** Tumor weight and **(I)** lymph node number from orthotopic rats exposed to hypoxia for 10 days. *p < 0.05 as compared with normoxia. n = 5 for each group.

We subsequently investigated the inhibitory effect of different durations of hypoxia exposure on lung cancer tumor growth in animals (Figure 1C). Four days after cancer cell injection subcutaneously, a tumor mass developed in the rats. After measuring the tumor volume and randomly grouping the animals, we then exposed the animals bearing tumor to hypoxia for 10 days and found that the tumor growth was also significantly inhibited as compared with normoxic controls (Figure 3A - E), showing significantly decreased tumor size during hypoxia (Figure 3A) and decreased tumor weight (Figure 3B) as well as the ratio of tumor weight to body weight (Figure 3C). Remarkably, almost all of the tumors from the normoxia group were larger than those from hypoxic animals (Figure 3D). Hematocrit was significantly increased in hypoxic animals (Figure 1E).

To further determine the effect of hypoxia on tumor progression, we let the tumors grow in rat for 7 days after cancer cell injection subcutaneously and then exposed the rats to hypoxia for another 7 days (Figure 1D). We found that exposure to hypoxia for 7 days also significantly inhibited the growth of lung cancer tumor (Figure 3F &3G).

In addition to the subcutaneous xenograft model, we used an orthotopic model of lung cancer to demonstrate the effect of hypoxia on the tumor growth and lymph node metastasis. We found that exposure to hypoxia for 10 days significantly inhibited the growth of lung cancer tumor (Figure 3I) and reduced lymph node metastasis (Figure 3J) in rats.

### Effect of normoxic recovery on hypoxic lung cancer tumor growth in rats

**Figure 4:**
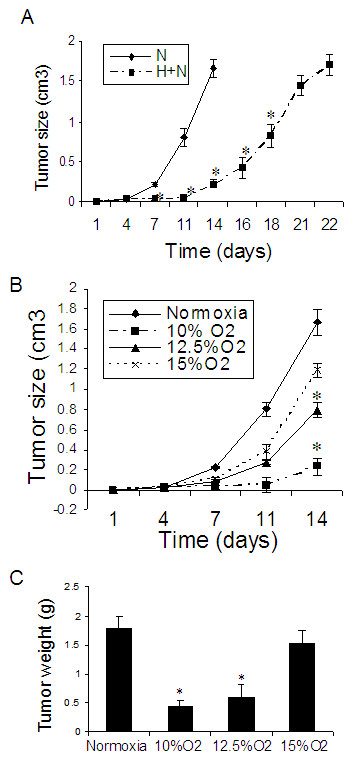
**Normoxia recovery and different levels of oxygen on lung cancer tumor growth in rats. Effect of normoxia recovery on hypoxia-induced reduction of lung cancer tumor growth (Figure 1E)**: (A) Tumor growth curve. Because large tumor mass was not allowed in living animals, we sacrificed the control rats when the tumor size reached over 1.5 cm. N = Rats under normoxia for entire experiment. H+N = Rats under hypoxia for 10 days and then under normoxia for another 8 days. **Effect of different levels of oxygen on lung cancer tumor growth (Figure 1C) (B & C)**: (B) Tumor growth curve and (C) Tumor weight. n = 5 for each group. *p < 0.05 as compared with normoxia.

We investigated if inhibition of the tumor growth was reversed after recovery under normoxia in the subcutaneous xenograft model (Figure 1E). After hypoxia exposure for 10 days, we moved the animals bearing lung cancer tumor back to normoxia. We found that the pattern of tumor growth was not changed immediately during normoxic recovery, showing continued inhibition of the tumor growth for several days (Figure 4A).

### Effect of different levels of oxygen on lung cancer tumor growth in rats

In order to determine which levels of oxygen most impacted tumor growth, we used 10%, 12.5% and 15% oxygen in the subcutaneous xenograft model. We found that 10% oxygen maximally inhibited lung cancer tumor growth after exposing animals to hypoxia for 10 days (Figure 4B &4C). There was no significant inhibition of the tumor growth under 15% oxygen.

### Exposure to hypoxia inhibited lung metastasis of Lewis lung carcinoma in mice

**Figure 5:**
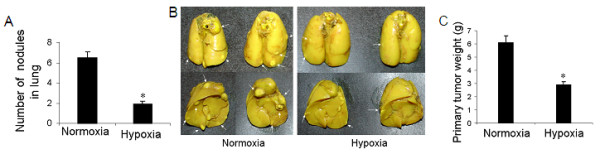
**Effect of hypoxia on lung metastasis of Lewis lung carcinoma in mice**. On the fourth day after cancer cell injection, the animals were placed under 10% hypoxia for 17 days (total of 3 weeks). After hypoxia exposure, the mice were sacrificed and lung metastasis nodules were counted. (A) Number of metastatic nodules in mouse lungs, (B) Representative lungs showing metastatic nodules the lungs. More and bigger metastasis nodules were seen in hypoxic mouse lungs than in normoxic lungs (arrows). (C) Quantitative data on primary tumor weight. *p < 0.05 as compared with normoxia. n = 10 mice for each group.

After 4 days of LLC1 cell injection subcutaneously, mice were exposed to hypoxia for 17 days. We found that hypoxia significantly inhibited lung metastasis of Lewis lung carcinoma in mice (Figure 5 A-C). Not only was the number of metastatic nodules in the lungs significantly reduced in hypoxic mice (Figure 5A), but also the size of the metastatic nodules was significantly decreased (Figure 5B). We also found a significant inhibition of primary tumor growth in hypoxic mice (Figure 5C).

### Effect of hypoxia-pretreatment on lung cancer tumor growth in mice

**Figure 6:**
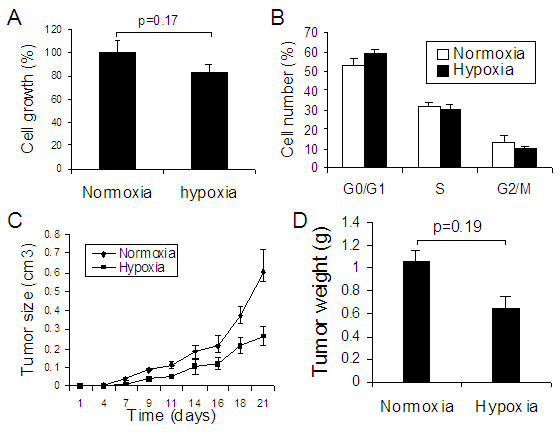
**Effect of hypoxia on lung cancer cell proliferation and cell progression in vitro and hypoxia-pretreatment on tumor growth in mice**. A549 cells were cultured in 0.5% hypoxia for 7 days and then harvested for cell proliferation assay and cell cycle progression analysis. In addition, the harvested cancer cells were inoculated into nude mice subcutaneously. After 21 days, the animals were sacrificed and the tumors were collected. (A) Cell growth assay and (B) cell cycle progression analysis *in vitro *(n = 9 for each group); (C) Tumor growth and (D) Tumor weight in mice. n = 7 for each group.

To determine if hypoxia-pretreatment affected tumor growth, we exposed A549 cells to 0.5% O_2 _for 7 days and then inoculated the cells into nude mice subcutaneously. First, we found that chronic hypoxia did not stimulate A549 cell proliferation and cell cycle progression *in vitro *(Figure 6A &6B). We then found that tumor growth from the hypoxia-pretreated lung cancer cells was also not stimulated as compared with the tumor from the cells without hypoxia-pretreatment (Figure 6C &6D).

### Differential effect of hypoxia on colon cancer

**Figure 7:**
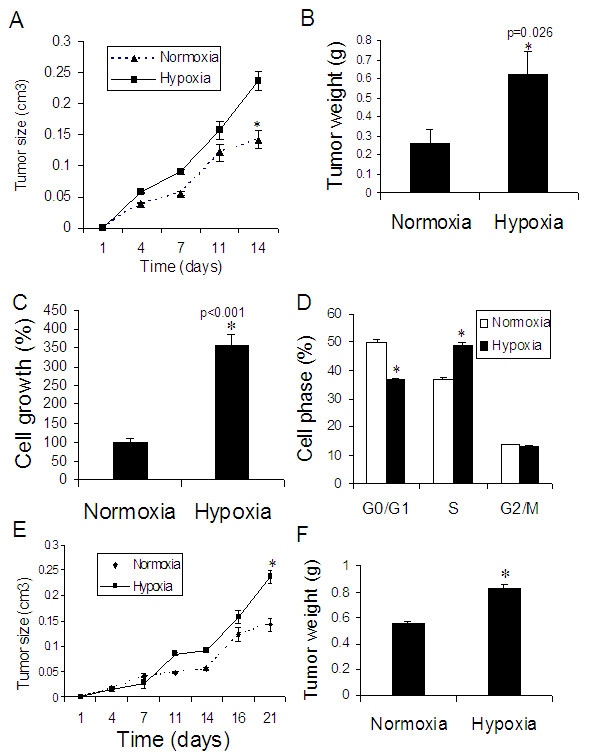
**Hypoxia exposure and colon cancer tumor growth.(A & B) Effect of hypoxia on colon cancer tumor growth (Figure 1C)**: Four days under normoxia after HCT116 colon cancer cell injection, animals were exposed to hypoxia (10% O_2_) for 10 days from xenograft model (protocols Figure 1C). (A) Tumor growth curve and (B) Tumor weight. *p < 0.05 as compared with normoxia. n = 6 rats for each group. **(C to F) Effect of hypoxia-pretreatment on colon cancer tumor growth**: HCT116 cells were cultured in 0.5% hypoxia for 7 days and then harvested for cell proliferation assay and cell progression analysis. In the meantime, the harvested cancer cells were inoculated into nude mice subcutaneously. After 21 days, the animals were sacrificed and the tumors were collected. (C) Cell growth assay and (D) cell cycle progression analysis *in vitro *(n = 9 for each group); (E) Tumor growth and (F) Tumor weight in mice. n = 5 for each group. *p < 0.05 as compared with normoxia.

To determine if long-term exposure to hypoxia impacted tumor progression similarly in different types of tumors, we investigated colon cancer by using the same conditions and procedures. In contrast to lung cancer, we found that long-term exposure to hypoxia significantly stimulated the growth of tumor from HCT116 cells (colon cancer tumor) in rats in the subcutaneous xenograft model (Figure 7A &7B). We also found that HCT116 cell growth and cell cycle progression were significantly stimulated under hypoxia (0.5% O_2_) (Figure 7C &7D) and hypoxia pretreatment significantly increased colon cancer tumor growth in mice (Figure 7E &7F).

### Hypoxia inhibited cell proliferation, increased apoptosis and decreased microvessel density in lung cancer tumors

**Figure 8:**
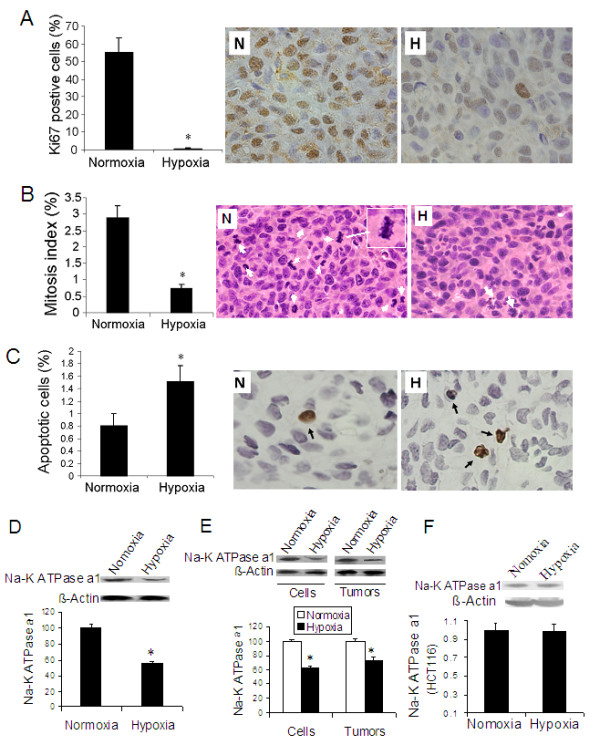
**Cell proliferation, apoptosis and Na+-K+ ATPase expression in lung cancer tumors.** Immunohistochemistry was used to evaluate cell proliferation and apoptosis. Mitosis was analyzed in slides with H & E stain. (A) Percent Ki67 positive cell number for cell proliferation; (B) Mitosis index and (C) Apoptosis. Left panel showing quantitative data and right panel showing representative micrographs. n = 5 for each group. **Expression of Na**^**+**^**-K**^**+ **^**ATPase α1 in lung cancer tumors and colon cancer tumors (D to F)**: Proteins were isolated from the tumors and Western blot was performed for analysis of Na^+^-K^+ ^ATPase α1 expression. Expression of Na^+^-K^+ ^ATPase α1 in lung cancer tumor in rats (D) and in A549 cells and in the lung cancer tumor from hypoxia-pretreated A549 cells in mice (E). (F) Na^+^-K^+ ^ATPase α1 expression in colon cancer tumor in rats (F). n = 3 for each group. Upper panels show representative images and lower panels show quantitative data, setting normoxia as 1. *p < 0.05 as compared with normoxia.

To investigate the mechanism underlying hypoxia-induced inhibition of lung cancer tumor growth, we examined cell proliferation and mitosis as well as apoptosis in tumor tissues of lung cancer grown in rats that exposed to 10% O_2 _for 10 days. We found a significant decrease in expression of Ki67 (Figure 8A), a cell proliferation marker, and in mitosis index (Figure 8B) in the tumor tissues from hypoxic animals. We also found a significant increase in apoptosis in the tumor tissues from hypoxic animals (Figure 8C).

### Hypoxia inhibited expression of Na^+^-K^+ ^ATPase in lung cancer tumors, but not in colon cancer tumors

To determine the signaling pathway that affected lung cancer growth under hypoxia, we examined expression of Na^+^-K^+ ^ATPase α1 subunit, which has been used as a marker of Na^+^-K^+ ^ATPase. We found a significant inhibition of Na^+^-K^+ ^ATPase α1 expression in tumors from hypoxic rats (Figure 8D) and in A549 cells exposed to hypoxia as well as in the tumor from the hypoxia-pretreated A549 cells grown in mice (Figure 8E). However, no significant change in Na^+^-K^+ ^ATPase α1 expression was observed in colon cancer tumor (Figure 8F).

### Effect of long-term exposure to hypoxia on HIF expression and microvessel density in lung cancer and colon cancer tumors

**Figure 9:**
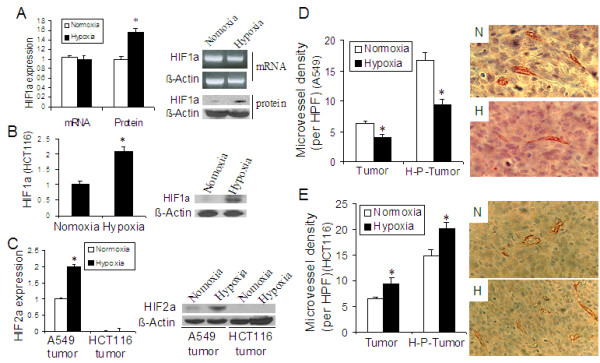
**HIF expression and microvessel density in lung and colon cancer tumors.(A to C) Expression of HIF1α and 2α:** Proteins were isolated from lung cancer tumors and colon cancer tumors and Western blot was performed for analysis of HIF expression. (A) Expression HIF1α mRNA and protein in lung cancer tumor; (B) Expression HIF1α in colon cancer tumor and (C) Expression of HIF2α protein in lung and colon cancer tumors. Left panels show quantitative data, setting normoxia as 1, and right panels show representative images of Western blot. n = 3 for each group. *p < 0.05 as compared with normoxia group. **(D & E) Microvessel density**: Immunohistochemical staining with CD31 antibody was performed to identify microvessels. Microvessel density in lung cancer tumors (D) and in colon cancer tumors (E). Left panels show quantitative data and right panels show representative images of immunohistochemistry (tumor from rats). Tumor = implanted tumor grown in rats. H-P-tumor = tumor from hypoxia-pretreated cancer cells grown in mice. Per HFP = average number of microvessels with CD31 positive staining/per high power field (× 200). n = 3 animals for each group. *p < 0.05 as compared with normoxia group.

We analyzed expression of HIF1α and HIF2α and used this as an indicator of effective exposure to hypoxia. We found significantly increased HIF1α protein expression in hypoxic lung cancer tumor (Figure 9A) and colon cancer tumor (Figure 9B). We also found a significant increase in HIF2α expression in the lung cancer tumor, but no HIF2α expression was detected in the colon cancer tumor (Figure 9C). In addition, tumor vascularisation was significantly affected by hypoxia exposure, but the results were significantly different in lung and colon cancer tumors. Compared with normoxic tumors, the microvessel density was significantly decreased in hypoxic lung cancer tumors (Figure 9D). However, the microvessel density was significantly increased in colon cancer tumors (Figure 9E). In addition, we measured HIF1α expression in the hypoxic tumors from the rats exposed to 12.5%O_2 _and 15%O_2 _found that 12.5%O_2 _significantly increased HIF1α expression, but no significant change in HIF1α expression was observed in the hypoxic tumor from animals exposed to 15%O_2_.

## Discussion

It has been reported that hypoxia plays a critical role in tumor progression. We in this study used different durations of hypoxia exposure, different concentrations of oxygen, different species of animals and different type of cancers to comprehensively investigate the effect of hypoxia on tumor progression. We first exposed rats to 10% oxygen for 14 days after cancer cell inoculation and found a significant inhibition of lung cancer tumor growth. This result indicated that hypoxia exposure inhibited lung cancer tumor development *in vivo*.

We further demonstrated that exposure to hypoxia could decrease tumor growth even after the tumor had already developed. A549 cells were inoculated into rats and grown for 4 days. On the fourth day, the tumor was palpable. We then placed the rats in the hypoxia chamber. The tumor growth was also inhibited after exposure to hypoxia. To confirm this result, we repeated this experiment three more times, used more than 20 rats for each group and obtained consistent results showing a significant inhibition of the lung cancer tumor growth in animals. Because hypoxic animals had a reduced body weight gain as compared with the nomoxic rats, we measured the ratio of tumor weight to body weight and obtained similar results, showing decreased ratio of tumor weight to body weight. Subsequently, we let the tumor grow in rats for a longer time (7 days) before exposure to hypoxia and found that the tumor growth was still decreased. In order to determine if the same condition of hypoxia affected other types of cancer growth, we placed rats with colon cancer tumor in the same chamber and found that the growth of colon cancer tumor was significantly stimulated. This result indicated that oxygen impacted lung cancer differently from other cancers. Interestingly, Kallimaki et al. [30] showed no significant change either in primary tumor growth or in lung metastasis in a transgenic mouse model of breast cancer after exposing the mice to hypoxia for 6 weeks. Kulish et al. [40] found that high-altitude hypoxia significantly inhibited growth of Guerin carcinoma and sarcoma 45 and increased the anti-tumor effect of chemotherapeutics on those tumors in albino rats. Terraneo et al. reported that exposing mice xenografted with a tumor of LNCaP prostate cancer under 10% O_2 _for 28 days significantly increased the tumor growth [29].

In previous studies, different concentrations of oxygen were used in different laboratories, from 2% O_2 _to 10% O_2 _[22,24,26,29]. To define the effect of different levels of oxygen on tumor growth, we used physiologically tolerated levels of 10%, 12.5% and 15% oxygen in this study. Our results showed that, except 15%, both 10% and 12.5% oxygen significantly decreased the tumor growth, in which 10% oxygen showed a greater inhibition than 12.5%. To determine if normoxia recovery increased or reversed tumor growth after hypoxia exposure, we housed rats with tumor in normoxia after removing them from the hypoxia chamber. We found that the inhibition of tumor growth of lung cancer persisted for almost a week after the rats were removed from hypoxia. In addition, we exposed A549 cells to 0.5% oxygen for 7 days and then inoculated the cells into mice. The mice were then housed under normoxia for three weeks, which avoided the influence of hypoxia on animals, such as reduced food intake and body weight loss. The growth of tumor from hypoxia-pretreated A549 cells were still not stimulated in those mice, further demonstrating that hypoxia is not a proliferative factor for the lung cancer tumor growth.

Hypoxia promotion of tumor progression in vivo has been found in other studies when different types of cancers have been studied [22-29], but the results have varied. Rofstad et al. found that hypoxia increased tumor progression of D12 and R-18 melanoma, including lung and lymph node metastasis in mice [22,23]. Zhang and co-workers [24] found increased lung metastasis of human fibriosarcoma from the cells pretreated with low oxygen in mice. Buchle and colleagues [25] found that tumor hypoxia influenced the number of metastatic lesions, but not the tumor volume in an orthotopic murine model for pancreatic cancer. Carines et al.[27,28] found that acute exposure to hypoxia significantly increased the number of positive lymph nodes, but not the lung metastasis nodules in human cervical carcinoma cell line (ME-180) in mice. A study by Kalliomaki et al. did not show significant change in lung metastasis of breast cancer in a transgenic mouse model [30]. In the present study, we used a rat orthotopic model of lung cancer to investigate lymphatic metastasis and a mouse model of Lewis lung carcinoma to study lung metastasis. We did not find any stimulating effect of hypoxia on either lymph node metastasis or lung metastasis. Our data thus indicated that hypoxia was also not a stimulating factor for metastasis of lung cancer.

We not only found decreased growth of lung cancer tumor *in vivo*, but also similar results *in vitro *in the growth of cultured lung cancer cells and in changes of proliferation markers in the tumor tissues. Hypoxia-induced inhibition of tumor progression of the lung cancer may be via an influence on Na^+^-K^+ ^ATPae. Previous studies have discovered impairment of Na^+^-K^+ ^pump function and of Na^+^-K^+ ^ATPase in lung alveolar type II cells [41,42]. Heerlein et al. [43] recently found that hypoxia exposure significantly decreased Na^+^-K^+ ^ATPase related oxygen consumption and indicated that inhibition of the Na^+^-K^+ ^ATPase by hypoxia contributed little to energy preservation in hypoxia. Hypoxia-induced inhibition of Na^+^-K^+ ^ATPase has been associated with promoting endocytosis of Na^+^-K^+ ^ATPase [44,45]. Mijatovic et al. [46] found an increase in expression of Na^+^-K^+^ATPase α1 subunit in clinical samples from patients with non small cell lung carcinoma as compared with normal lung tissues. They also observed that reduction of Na^+^-K^+ ^ATPase α1 expression by a siRNA in A549 cells resulted in markedly decreased proliferation of the cells. In addition, Xu et al. [47] recently reported that the expression of Na^+^-K^+ ^ATPase α1 was higher in human hepatocellular carcinoma than in normal liver tissues and that a siRNA for Na^+^-K^+ ^ATPase α1 inhibited proliferation of human HepG2 liver cancer cells. To demonstrate if hypoxia-induced inhibition of the lung cancer tumor progression was involved in Na^+^-K^+ ^ATPase dysfunction, we investigated expression of Na^+^-K^+ ^ATPase α1 subunit, which has been used as a marker of Na^+^-K^+ ^ATPase, in the lung cancer tumors. We found significantly decreased expression of Na^+^-K^+ ^ATPase α1 not only in the lung cancer tumor from the rats exposed to hypoxia, but also in the tumor from hypoxia-pretreated A549 cells in mice. Because the mice with the tumor from hypoxia-pretreated cancer cells were housed under normoxia, this result indicated continued inhibition of Na^+^-K^+ ^ATPase expression by hypoxia; even after the tumor grew under normoxia for 3 weeks. These results demonstrated that hypoxia-induced inhibition of tumor progression of the lung cancer in animals was associated with a decrease in Na^+^-K^+ ^ATPase, which might result in a decrease in oxygen consumption and consequently in inhibition of cell functions, including cell division. Interestingly, the expression of Na^+^-K^+ ^ATPase α1 in colon cancer tumor was not significantly changed under hypoxia, which might be one of the reasons that the growth of colon cancer tumor was not inhibited under hypoxia.

Hypoxia-inducible factors (HIF1α and HIF2α) mediate cellular response to hypoxia, which are usually induced under hypoxic condition. Over expression of HIF1α has been thought to be positively related to tumor progression. However, we found a significant increase in HIF1α protein expressions in hypoxic lung and colon cancer tumors, but the tumor progression of lung cancer was repressed. Actually, conflicting results have been observed in other investigations although mainstream research has shown that HIF1α promotes tumor progression. Volm et al. found that patients with HIF1α-positive non-small cell lung carcinoma had significantly longer median survival times than those patients with HIF1α-negative carcinoma [48]. Beasley and colleagues reported that expression of HIF1α in surgically treated patients with head and neck squamous cell carcinoma was associated with improved disease-free survival and overall survival [49]. Fiorenzo et al. recently reported that inhibition of the HIF1α by RNA interference did not decrease tumor growth in human glioblastoma multiforme cells [50]. Besides HIF1α, we also found increased expression of HIF2α in the lung cancer tumor. However, no HIF2α expression was detected in the colon cancer tumor from HCT116 cells. HIF2α has been reported to play a similar role to HIF1α. However, Imamura et al. recently reported different result. They found that deficiency of HIF2α stimulated SW480 colon cancer cell tumor growth and suggested that HIF2α appeared to restrain tumor growth [51]. An absent or minimal expression of HIF2α under hypoxia was observed in other colon cancer cells [51]. Therefore, the absent expression of HIF2α might be another reason that long-term exposure to hypoxia enhanced the growth of colon cancer tumor. In addition, it has been thought that over expression of HIF1α and HIF2α is positive related to tumor angiogenesis. We thus examined microvessel density in both lung and colon cancer tumors and found significantly reduced microvessel density in lung cancer tumor. However, significantly increased microvessel density in colon cancer tumor was observed. Therefore, HIF1α and HIF2α may be playing different roles in the progression of lung cancer tumor and colon cancer tumor during hypoxia.

This study demonstrated that long-term exposure to hypoxia suppressed lung tumor progression in different animal models and found a decrease in Na^+^-K^+ ^ATPase expression in hypoxic lung cancer tumors. However, some limitations have to be pointed out. For example, we only used A549 cell line and HCT116 cell line; and the precise relationship between the hypoxia-induced expressions of HIFs and the down regulation of Na^+^-K^+ ^ATPase has not been determined. Because HIF is the major mediator in cellular adaption to hypoxia, increased HIF1α and HIF2α might be involved in mediating the endocytosis of Na^+^-K^+ ^ATPase. However, a recent study [52] showed that von Hippel Lindau protein (pVHL) participated in hypoxia-mediated degradation of plasma member of Na^+^-K^+ ^ATPase, but knock down HIF1α and HIF2α by shRNA did not prevent Na^+^-K^+ ^ATPase degradation in A549 cells during hypoxia, which suggested that HIFs were not required for Na^+^-K^+ ^ATPase degradation. In addition, we do not know why lung cancer cells and colon cancer cells respond to hypoxia so differently. It might be possible that lung cells, instructed to resist relatively high and oscillating PO2 in the alveoli, are less hypoxia-sensitive than colon cancer cells, which would shift the balance between pro-cancer (HIF expression) and anti-cancer (apoptosis, endocytosis) mechanisms toward anti-cancer, whereas the same degree of hypoxia shifts the same balance to the other end in cells instructed to live in relatively more hypoxic environments. Those questions will be the subject of our future work.

## Conclusions

In summary, we in this study found that long-term exposure to hypoxia significantly inhibited tumor progression of lung cancer in animals and significantly decreased Na^+^-K^+ ^ATPase α1 expression in the tumors. This study demonstrated that hypoxia is not a promoting factor for tumor progression of lung cancer from A549 cells and that decreased expression of Na^+^-K^+ ^ATPase may be involved in hypoxic inhibition of tumor progression of the lung cancer. The results from this study provide new insights into the role of hypoxia in tumor progression and therapeutic strategies for cancer treatment,

## Competing interests

The authors declare that they have no competing interests.

## Authors' contributions

LY initiated and designed this study, performed experiments and wrote manuscript. CAH advised on the project and revised manuscript. All authors read and approved the final manuscript.

## Pre-publication history

The pre-publication history for this paper can be accessed here:

http://www.biomedcentral.com/1471-2407/11/331/prepub
